# Role of Ethyl Pyruvate in Systemic Inflammatory Response and Lung Injury in an Experimental Model of Ruptured Abdominal Aortic Aneurysm

**DOI:** 10.1155/2014/857109

**Published:** 2014-01-19

**Authors:** Zerrin Pulathan, Gökalp Altun, Doğuş Hemşinli, Ahmet Menteşe, Esin Yuluğ, Ali Civelek

**Affiliations:** ^1^Department of Cardiovascular Surgery, Faculty of Medicine, Karadeniz Technical University, 61080 Trabzon, Turkey; ^2^Depertment of Biochemistry, Faculty of Medicine, Karadeniz Technical University, 61080 Trabzon, Turkey; ^3^Depertment of Histology and Embryology, Faculty of Medicine, Karadeniz Technical University, 61080 Trabzon, Turkey

## Abstract

*Objectıve*. The purpose of this study is to evaluate the effect of ethyl pyruvate (EP) on systemic inflammatory response and lung injury in an experimental rat model of ruptured abdominal aortic anurysm (RAAA). *Methods*. Anaesthetized 30 Sprague-Dawley male rats were randomized to sham (Sh *n* : 6) (Sh + EP *n* : 6) or shock and clamp (S/C) groups (S/C *n* : 9) (S/C + EP *n* : 9). In the S/C and S/C + EP groups, hemorrhagic shock, lower torso ischemia, and reperfusion were created, S/C group was given 1 mL saline and S/C + EP group was given 40 mg/kg EP. At the end of reperfusion process some biochemical and histological parameters were studied in serum and lung tissues. *Results*. An increase was observed in all parameters except interleukin-6 (IL-6) in the S/C group in comparison to the sham groups. In the S/C + EP group, serum myeloperoxydase (MPO), malondialdehyde (MDA), and tumor necrosis factor alpha (TNF-**α**) as well as lung MPO and MDA values decreased significantly (*P* < 0.016). In the lung tissues, histological injury scores and lung tissue wet/dry ratio were significantly decreased in the S/C + EP group as compared to the S/C group (*P* < 0.016). *Conclusions*. Ethyl pyruvate may reduce systemic inflammatory response and lung injury which resulted from shock and ischemia/reperfusion in an experimental model of RAAA.

## 1. Introduction

Ruptured abdominal aortic aneurysm (RAAA) accounts for 1-2% of deaths in the population older than 65 years of age. However endovascular therapy (EVAR), which is increasingly being performed, has decreased the mortality rate to 20–25% from 50–70% encountered in the open repair (OR) performed for the treatment of RAAA. Today, open repair is the most frequently (80%) performed method because of anatomical inconvenience, such as short neck and poor iliac arteries, or inadequate team and equipment in the vascular surgery centers [1].

Whilst ischemia and reperfusion occur only in the lower torso with the clamping and declamping during surgical treatment of intact aneurysm, preclamping hemorrhagic shock and related impaired tissue perfusion accompany the ischemia and reperfusion of the lower torso during surgical treatment of ruptured aneurysm. This complex situation leads to systemic inflammatory response syndrome (SIRS) and multiorgan failure (MOF) by causing local or distant organ damage such as lungs, liver, and heart and enhances the mortality rate in the surgical treatment of RAAA. Lindsay et al. created an experimental model and demonstrated that supramesenteric aortic clamping and hemorrhagic shock, each of which is unable to cause lung damage individually, caused distant organ damage when they are together, as in the surgical treatment of RAAA, and they used this model in numerous experimental studies [2]. Ischemia-reperfusion injury causes distant organ damage by generating systemic inflammatory response [3].

Ethyl pyruvate (EP) is a simple ester derivative of pyruvic acid. In many animal models, in which various critical diseases including endotoxemia, sepsis, hemorrhagic shock, burn injury, pancreatitis, ileus, and myocardial, mesenteric and hepatic ischemia/reperfusion injuries were modeled; EP was demonstrated to reduce organ damage and have favorable effect on survival. EP is an effective anti-inflammatory agent and shows its efficacy by decreasing the secretion of proinflammatory proteins such as TNF-*α* and IL-6. EP has also been demonstrated to be an effective free oxygen radical scavenger [4–7]. Efficacy of ethyl pyruvate has not been demonstrated previously in an experimental model in which shock, ischemia, and reperfusion are found together.

The study aimed at investigating the effect of ethyl pyruvate on inflammatory response and lung injury by creating a rat model of ruptured abdominal aortic aneurysm.

## 2. Materials and Methods

### 2.1. Animal Care

The present study was performed in 30 Sprague-Dawley male rats with a mean weight of 430 ± 45 gram after the approval of Karadeniz Technical University, Animal Experiments Local Ethics Committee. The rats were kept at a room temperature of 23°C in 12/12 hours dark/light cycle and fed with standard chow and water until the study day. All rats were famished for 12 hours prior to the experiment and they were given only water

### 2.2. Solutions

In order to administer in the treatment groups, ethyl pyruvate (Sigma-Aldrich, Cat no. E4, 780-8) was prepared in Ringer's solution as per 1 mL would include 20 mg ethyl pyruvate. Ringer's Lactate solution was used in the control groups approximately in the same amount with EP. Ringer's solution includes 129.3 mEq/L Na^+^, 5.0 mEq/L K^+^, 112 mEq/L Cl^−^, and 4 mEq/L Ca^2+^ and its osmolality is 275.5 mOsm/L (Polypharma, Turkey).

### 2.3. Experimental Design

Intraperitoneal ketamine (50 mg/kg) and xylazine (10 mg/kg) were used for the anesthesia of the rats. The anesthesia was maintained with intermittent ketamine administration allowing spontaneous respiration. Rats were randomized in two groups as sham (Sh) and shock/clamp (S/C); each group was redivided into two groups (Sh (*n* = 6), Sh + EP (*n* = 6), S/C (*n* = 9), and S/C + EP (*n* = 9)), in the way that the operator would be blind for the treatment. Sh and Sh + EP groups underwent no surgical procedure except for aortic exploration, whereas S/C and S/C + EP groups underwent shock for 60 minutes, ischemia for 60 minutes, and reperfusion for 120.  Table 1 summarizes the experimental groups and the course of experiment.

Cutdown was performed and the right internal jugular vein was cannulated for the venous access, and the right carotid artery was cannulated using number 22 cannula (Novacath, Medipro, Co., Istanbul, Turkey) to monitor mean arterial pressure (MAP). Heart rate, MAP, rectal temperature, and respiratory rate were monitored (Nikon Kohden BSM-4113). In the course of experiment, 3 mL/kg/h of saline (0.9% NaCl) infusion (Perfusor Compact S, Brown) was done to meet the insensible losses. Rectal temperature was kept around 36.5°C using heat lamb. Midline laparotomy was performed in all groups and abdominal aorta was isolated at the level of proximal side of superior mesenteric artery and iliac bifurcation. Laparotomy was closed using 5/0 Prolene suture in the sham groups and the rats were kept under anesthesia for 4 hours. In the S/C groups, blood sample was drawn through the carotid artery cannula into the heparinized plastic injector (500 U Heparin) (Nevparin, 5000 U/mL, Mustafa Nevzat, Turkey) after the monitoring and stabilization periods, and shock was created keeping the MAP at 50 mmHg for 60 minutes and aneurysm rupture was simulated; the blood was stored at room temperature. The amount of blood obtained from the rats was calculated not to exceed 30% of the total blood volume. Equal amount of Ringer's solution was given to the S/C and Sh groups at the end of 60-minute hemorrhagic shock and equivalent period, respectively, whereas Sh + EP and S/C + EP groups received intraperitoneal (ip) 40 mg/kg EP. Abdominal aorta was explored through the midline laparotomy. All the groups underwent systemic heparinization by intravenous heparin given at a dose of 250 U/kg. In the S/C and S/C + EP groups, lower torso ischemia was created by clamping abdominal aorta separately at the level of superior mesenteric artery and iliac bifurcation using microvascular clamps. Half of the blood sample taken at that time was reinfused via venous route, and surgical x-clamp and resuscitation were simulated. Following 60-minute ischemic period, all of the remaining blood was reinfused just before the clamp was removed. After the clamps were removed, the abdomen was closed and the rats were kept in reperfusion for 120 minutes. The MAP was kept at 100 mmHg during reperfusion period by administering additional saline solution when needed. The amount of fluid administered in the course of experiment was recorded.

At the end of experimental period, all rats were sacrificed by drawing blood through the carotid artery. Hilar regions of both lungs were clamped and removed; a part of the left lung tissue was stored at −80°C as frozen for biochemical analyses, whereas the other part was fixed with formaldehyde for histopathological examinations. Right lungs were reserved for the calculation of the wet/dry weight ratio.

### 2.4. Laboratory Analysis

The serum levels of malondialdehyde (MDA), myeloperoxydase (MPO), tumor necrosis factor-alpha (TNF-*α*), interleukin-6 (IL-6), ischemia modified albumin (IMA), and blood gases were measured in the blood samples.

MDA and MPO were measured in the lung tissue and histopathological examination was performed.

#### 2.4.1. MDA Measurement

The red color that resulted from the reaction between MDA, a lipid peroxidation product, and thiobarbituric acid (TBA) was measured spectrophotometrically at 532 nm light. Plasma level was calculated as nanomol/mL (nanomoles per milliliter) [8].

#### 2.4.2. MPO Measurement

Serum MPO levels were assessed using enzyme-linked immunosorbent assay (ELISA) kit (Hycult biotech, Catalog number HK105, The Netherlands). Absorbance of the samples was measured by VERSA brand (designed by Molecular Devices in California, USA) microplate reader at 450 nm. Results were given as nanogram/mL [9].

#### 2.4.3. Serum TNF-*α* Measurement

Serum TNF-*α* level was measured using enzyme-linked immunosorbent assay (ELISA) kit (Bender MedSystems, Catalog number BMS622, Vienna, Austria). Absorbance of the samples was measured by VERSA brand (designed by Molecular Devices in California, USA) microplate reader at 450 nm. Results were given as picogram/mL [10].

#### 2.4.4. Serum IL-6 Measurement

Serum IL-6 levels were measured by a method similar to that of TNF-*α*. Results were given as picogram/mL.

#### 2.4.5. Serum IMA Measurement

Reduced Cobalt-to-albumin binding capacity was evaluated by rapid and calorimetric assessment method developed by Bar-Or et al. Once blood samples were taken, serum and plasma specimens were prepared by centrifuging at 1.800 ×g for 15 minutes. The specimens were put into Eppendorf tubes and stored at −80°C until analysis. Reduced cobalt-to-albumin- binding capacity (IMA level) was analyzed using rapid colorimetric method. The results were given as absorbance units (ABSU) [11].

#### 2.4.6. Blood Gas Measurement

At the end of experimental period, 0.5 mL of blood was drawn from the carotid artery to remove the probable serum residues and was disposed. Subsequently, approximately 1.5 mL of arterial blood sample taken into heparinized injector was transferred into the cartridge and blood gasses were measured (IRMA TRU point Blood Analysis System).

#### 2.4.7. MDA Measurement in the Lung Tissue

A piece of lung tissue was used to measure MDA levels. The sample was minced and homogenized by an Ultra-TurraxT25 homogenizer (Janke and Kunkel IKA) in an ice-cold 1.15% KCl solution containing 0.05% Triton X-100. Tissue MDA levels were determined using the method described by Uchiyama and Mihara. Tetramethoxypropane was used as a standard. The MDA levels were calculated in nanomoles per gram of wet tissue [12].

#### 2.4.8. MPO Measurement in the Lung Tissue

Tissue MPO levels were assessed using enzyme-linked immunosorbent assay (ELISA) kit (Hycult biotech, Catalog No. HK105, The Netherlands). Absorbance of the samples was measured by VERSA brand (designed by Molecular Devices in California, USA) microplate reader at 450 nm. Results were given as nanogram/mL per gram of tissue.

#### 2.4.9. Evaluation of the Lung Edema (Wet/Dry Ratio)

As the experiment was finalized, sternotomy was performed and the right lung was removed by clamping at the hilus, separated from the surrounding tissues and weighed using microbalances. It was reweighed after being stored at 70°C for 48 hours. Wet/dry weight ratio was calculated, and an increase was interpreted in favor of lung edema.

#### 2.4.10. Histopathological Examination of the Lung Tissue

Following the finalization of the experiment, tissue samples taken from approximately the same lung sections in all rats were separately kept in numbered storages containing 10% of neutral formaldehyde solution for histopathological examination. The bloody solution was changed after 30 minutes, and the tissues were fixed with 10% neutral formaldehyde solution for 48 hours. Tissues were dehydrated by passing through the graded series of alcohol and embedded into paraffin after being made pellucid. The paraffin blocks were cut in 5 micrometer (*μ*m) thicknesses by a microtome (Leica RM2255, Japan) and placed onto numbered slides. The sections on the slides were placed in a woven basket, kept in the incubator at 50°C for 30 minutes, and then deparaffinized and dehydrated with alcohol series. These sections were stained with Hematoxylin and Eosin (H&E); the preparations were dehydrated with alcohol series and xylene; entellan was dripped on the preparations and covered with lamella. Histopathological examination was done by an experienced histologist blind for the study groups. For the injury scoring of the lung tissues, 5 different areas in the lung preparations of each group were semiquantitatively evaluated with high magnification (400x) according to the criteria defined below [13]. Microscopic scoring criteria of lung injury were graded between 0 and 4. Grade 0: normal lung morphology, Grade 1: mild intra-alveolar edema and mild inflammatory cell infiltration, Grade 2: moderate intra-alveolar edema and moderate inflammatory cell infiltration, Grade 3: severe alveolar edema, severe inflammatory cell infiltration, and focal hemorrhage, and Grade 4: disseminated inflammatory cell infiltration and destruction in alveolar structure.

### 2.5. Statistical Analysis

Statistical analysis was performed using SPSS 15.0. Kruskal-Wallis variance analysis (the Mann-Whitney *U* test with Bonferroni correction as post hoc) was used to compare the groups. The results were expressed as mean ± standard deviation (SD). Statistical significance was considered to be *P* < 0.016.

## 3. Results

The results of serum MPO, MDA, TNF-*α*, IL-6, IMA, and others are given as mean ± standard deviation in Table 2.

### 3.1. Serum MPO

Whilst MPO level was 81.82 ± 34.21 and 96.91 ± 49.32 ng/mL in the Sh and Sh + EP groups, respectively, it increased by 4 folds, reached to 383.85 ± 38.19 ng/mL in the S/C group, and decreased to 198.33 ± 69.16 ng/mL in the S/C + EP group. These results indicated no statistical difference between groups Sh and Sh + EP, whereas MPO was significantly increased in the S/C group versus the sham group (*P* = 0.002) and statistically significantly decreased in the S/C + EP group versus the S/C group. This result suggests that ethyl pyruvate suppresses neutrophil activation, which was enhanced by shock, ischemia, and reperfusion.

### 3.2. Serum MDA

Lipid peroxidation is one of the reactions caused by free oxygen radicals, and MDA is one of the end products of lipid peroxidation. MDA levels were increased by two folds in the S/C group versus the sham groups and reached to 2.95 ± 1.45 nmol/mL (*P* = 0.000) but decreased to 1.96 ± 0.61 nmol/mL in the S/C group that received EP (*P* = 0.004).

### 3.3. TNF-*α*

TNF-*α*, which is an important proinflammatory cytokine, was significantly increased at the end of clamping and reperfusion periods as compared to the sham groups. Comparing to the S/C group, TNF-*α* concentration was significantly suppressed in the S/C + EP group and decreased to 188.76 ± 56.55 pg/mL from 262.71 ± 18.24 pg/mL (*P* = 0.004).

### 3.4. Interleukin-6

IL-6 level was minimally increased in the S/C group versus the sham groups, but no statistically significant difference was found between the groups (whilst it was 196.53 ± 52.22 pg/mL in the S/C group, it was 183.98 ± 56.75 pg/mL in the S/C + EP group, *P* = 0.48).

### 3.5. IMA

This marker, which depends on the measurement of the amount of circulating albumin modified due to ischemic stress, was increased by two folds from 0.42 ± 0.3 ABSU in the sham group to 0.84 ± 0.43 ABSU in the S/C group (*P* = 0.003) but was not significantly decreased in the S/C + EP group (*P* = 0.22).

### 3.6. Lung MDA and MPO

Lung tissue MDA level was significantly increased to 522.5 ± 81.12 nmol/g in the S/C group from 454.29 ± 35.2 nmol/g in the sham groups (*P* = 0.000) and decreased to 499.14 ± 43.2 nmol/g in the S/C + EP group (*P* = 0.001).

Likewise, lung MPO level increased to 6541.1 ± 613.4 ng/mL in the S/C group from 5636.77 ± 404.7 ng/mL in the sham group and decreased to 6019.12 ± 394.4 ng/mL in the S/C + EP group (*P* = 0.014).

### 3.7. Lung Wet/Dry Weight Ratio

There was no significant difference between the sham groups (*P* = 0.748) in terms of lung wet/dry weight ratio, but it was increased in the S/C group. The wet/dry weight ratio of the lung tissues decreased to 3.77 ± 0.74 in the group that received ethyl pyruvate from 5.55 ± 0.45 in the SIR group and lung edema was significantly reduced (*P* = 0.001).

### 3.8. Blood Gase

Blood gas analysis of the groups revealed increase in the acidosis and base gap in the S/C and S/C + EP groups versus the sham groups. pH decreased to 7.20 ± 0.09 in the S/C group from 7.38 ± 0.04 in the sham groups (*P* = 0.014); although it decreased to 7.33 ± 0.07 in the S/C + EP group, it was not statistically significant (*P* = 0.4). Whilst base gap (BG) was 0.43 ± 1.38 in the Sh group, it was −9.38 ± 5.04 in the S/C group (*P* = 0.01) and decreased to −5.64 ± 2.8 in the S/C + EP group, but this improvement was not significant (*P* = 0.48). No difference was found between the groups in terms of PO_2_, PCO_2_, and HCO_3_ values. Blood gas values are summarized in Table 3.

### 3.9. Lung Injury

**Figure 1:**
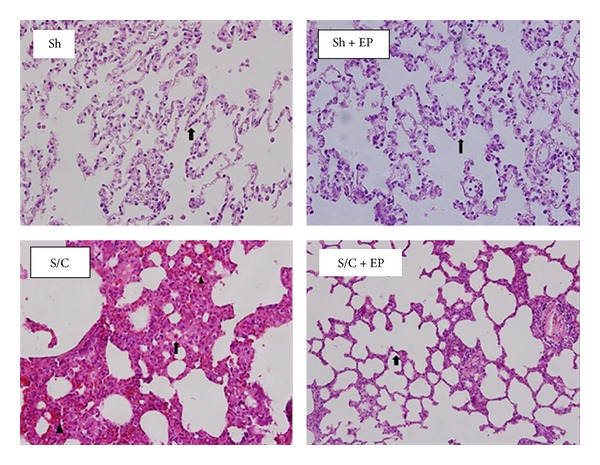
(Sh, Sh + EP) Normal histopathological examination of the lung tissues in Sh and Sh + EP groups. (S/C) Diffuse intra-alveolar edema (↑), intra-alveolar hemorrhage (▲), and leukocyte infiltration in the S/C group. (S/C + EP) Moderate intra-alveolar edema and alveolar epithelial thickening (↑) were observed in the S/C group that received EP. (Hematoxylin and Eosin, [H&E] ×200).

Histopathological examination of the lung tissues revealed normal lung tissue in the sham groups but diffused intra-alveolar edema, intra-alveolar hemorrhage, and leukocyte infiltration in the S/C group. Moderate intra-alveolar edema and alveolar epithelial thickening were observed in the S/C group that received EP (Figure 1). It were observed that histopathological injury score of the lung tissue was increased by 5 folds and reached to 2.66 ± 0.7 in the S/C group from 0.5 ± 0.8 in the sham group but decreased to 1.44 ± 0.72 in the S/C group given EP (*P* = 0.012).

### 3.10. Hemodynamic Alterations and Fluid Resuscitation

Whilst the mean arterial pressure (MAP) was stable in the sham groups over the course of experiment, the amount of saline given to achieve the baseline level of MAP was low in these groups.

After the baseline level was achieved, the MAP was kept around 50 mmHg in the S/C and S/C + EP groups in accordance with the experiment protocol. The amount of blood taken from the rats was 6.7 ± 0.5 mL for each group. MAP was higher in the S/C groups than the sham groups during 60-minute aortic clamping period after the shock period. Whilst MAP was 85.9 ± 4.2 mmHg in the sham groups, it was 132.7 ± 5.9 mmHg in the S/C groups (*P* = 0.0012).

**Figure 2:**
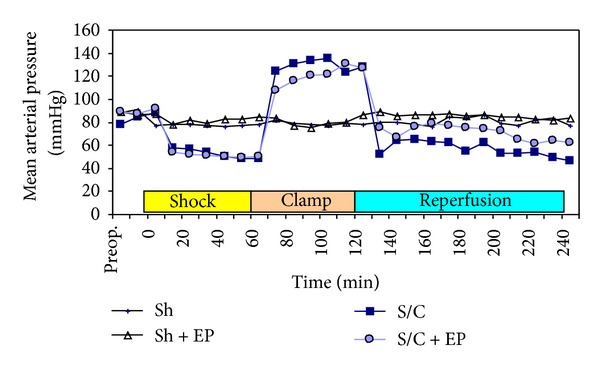
Mean arterial blood pressure during the experiment. Values are mean ± SEM.

During reperfusion phase, MAP decreased rapidly and became lower than that in the sham groups; decrease in MAP in the S/C group was more remarkable through the end of 120-minute reperfusion period (Figure 2). Whilst MAP was 51.34 ± 6.3 mmHg in the S/C group on the 120th minute of reperfusion period, it was 64.91 ± 5.8 mmHg in the S/C + EP group (*P* = 0.011).

Total amount of extra fluid given in addition to the maintenance fluid was 5.2 ± 0.7 mL in the S/C group, whereas it was 3.4 ± 0.6 mL in the S/C + EP group (*P* = 0.0018).

## 4. Discussion

In the present study, we investigated whether ethyl pyruvate reduces systemic inflammatory response and lung injury that result from shock, ischemia, and reperfusion in the surgical treatment of the rat model of ruptured abdominal aortic aneurysm. Systemic inflammatory response syndrome (SIRS) and related multiorgan failure are the most important causes of high mortality following surgical treatment of ruptured abdominal aortic aneurysm. Whilst the prevalence of multiorgan failure is 3.8% after surgical treatment of intact aneurysms, it is 64% after RAAA [14].

Unlike intact aneurysm, total body ischemia, which varies depending on the duration and deepness of hemorrhagic shock, occurs in ruptured aneurysms and lower torso ischemia and reperfusion, which result from aortic clamping, accompany this situation in the course of surgery. Although many systems including complement, coagulation, fibrinolytic, and kallixrein cascades are activated during inflammatory response triggered together by hemorrhagic shock, ischemia, and reperfusion, leukocytes play the most important role. Activation and interaction of leukocytes and endothelial surface initiate the cytokine release, endothelial microvascular permeability is increased, and transendothelial neutrophil migration occurs. Endothelial damage leads to endothelial cell swelling, capillary leak, edema, and organ dysfunction. This situation known as systemic inflammatory response is followed by multiple organ failure including the lungs and the kidneys. In many of the ischemia and reperfusion studies, some biochemical analyses are performed including cytokine levels such as TNF-*α* and IL-6 that indicate systemic or local organ damage, neutrophil activation marker MPO, MDA that indicates tissue hypoxia and free radical-induced lipid peroxidation, and ischemia-specific marker IMA; and damaged distant organ is examined both histopathologically and functionally.

Pyruvic acid, which is a carboxylic acid with three carbons, is the end product of glucose metabolism as a member of aliphatic amino acids. The reaction between pyruvate and coenzyme A results in Acetyl CoA, which is a rate-limiting step in the oxidative metabolism of glucose by thiocarboxylic acid. Although pyruvate is a metabolic fuel, it also functions as an endogenous oxygen radical scavenger.

Ethyl pyruvate (EP) is a simple aliphatic ester derivative of pyruvic acid. In various animal experiments, it was demonstrated to be an effective anti-inflammatory agent by inhibiting proinflammatory signal pathways such as NF-*κ*B and p38 mitogen-activated proteinkinase. Moreover, it reduces (downregulates) the secretion of proinflammatory proteins such as TNF, IL-6, and high mobility group box 1 (HMGB1), and was demonstrated to be an effective reactive oxygen species (ROS) scavenger [15, 16]. Since ethyl pyruvate is stabilized by forming enolate with calcium, it turns into a more stable compound when dissolved in Ringer's lactate solution. Therefore, many of the studies use ethyl pyruvate dissolved in Ringer's lactate. Cruz e al. performed ischemia-reperfusion study using various solutions of pyruvate and found that ethyl pyruvate to be more effective than other pyruvate derivatives [15]. In the present study, we as well used this solution and additionally formed a sham group with Ringer's lactate similar to the literature.

Lindsay et al. conducted an experimental study and created shock, ischemia, and reperfusion, and this method was called experimental model of ruptured abdominal aortic aneurysm [2]. In this study, shock, ischemia, and reperfusion procedures were performed in the experimental groups in different periods; aortic clamp was applied to different places as supramesenteric and inframesenteric, and pulmonary permeability index and MPO activity in the lung tissue were measured in all groups and were compared with each other. As a result, it was determined that 1-hour shock, 2-hour supramesenteric clamping, and 2-hour reperfusion periods provide the highest increment in pulmonary permeability index and lead to neutrophil accumulation. Later on, this model has been used in many studies. In the present study, we as well investigated systemic inflammatory response, lung injury, and the effect of ethyl pyruvate on this injury, in a model of ruptured abdominal aortic aneurysm, in which shock, ischemia, and reperfusion could be stimulated all together. We measured serum MDA, MPO, TNF-*α*, IL-6, IMA, and blood gas values, as well as MDA, and MPO values in the lungs. In addition, we histopathologically examined the lung tissue and assessed the lung edema.

Lipid peroxidation is one of the reactions caused by free oxygen radicals, and MDA is one of the end products of lipid peroxidation. Measurement of MDA level in the plasma and tissues gives information about free oxygen radicals. In a substantial proportion of experimental IR studies, information about lipid peroxidation in the serum and tissues was obtained by measuring serum MDA values using various methods. Jan et al. measured MDA values as a parameter to investigate whether ischemic preconditioning performed prior to shock reduces lung injury [17]. In the present study, it was observed that MDA values were remarkably increased both in the serum and in the lung tissues of the S/C group versus the sham group and that radical production was suppressed in the S/C + EP group. Ethyl pyruvate remarkably suppressed lipid peroxidation and accordingly free radical production. Taylor et al. created a rat model of myocardial ischemia-reperfusion and demonstrated that ethyl pyruvate reduces lipid peroxidation in the ischemic myocardial tissue [18]. Cruz et al. found that ethyl pyruvate significantly reduces hepatic MDA level in the visceral ischemia-reperfusion model [15]. Results of the present study are consistent with these two studies.

MPO, which is a peroxidase enzyme and mostly found in the neutrophils, is a lysosomal protein stored in the azurophilic granules of the neutrophils. Measurement of serum and tissue MPO values gives information about neutrophil activation. In the present study, increased MPO activity both in the systemic circulation and in the lungs due to S/C application was suppressed by EP. Wang et al. and Luan et al. demonstrated that MPO activity in the ileum was suppressed by EP in the extrahepatic cholestasis and in acute pancreatitis, respectively [19, 20]. The results of the present study are consistent with the results of above-mentioned authors. There is no evidence that ethyl pyruvate suppresses the neutrophil activation both in the systemic circulation and in the distant organ. With this regard, the present study is the first.

Tumor necrotizing factor-*α* is a very important proinflammatory cytokine that weighs 17 kDa, secreted from activated macrophages, monocytes, t-lymphocytes, killer cells, and fibroblasts, and binds to the cell membrane via specific receptors. In the present study, serum TNF-*α* levels were significantly increased in the S/C group and suppressed by EP administration. Cai et al. used ethyl pyruvate as the resuscitation fluid in hemorrhagic shock and demonstrated that TNF-*α* level was decreased and survival was increased in the rats [21]. This suggests that systemic inflammatory response is substantially suppressed by EP. Kung et al. created lung injury in rats by lipoteichoic acid and reported that ethyl pyruvate suppresses TNF-*α* and prevents lung injury via anti- inflammatory effect and this effect is dose-dependent [22]. In an experimental study in which Shahani performed the same RAAA model, it was published that TNF-*α* level was 10 times increased in the myocardial tissues of the rats versus the control groups and cardiac functions were improved by immune neutralization of TNF-*α* [23]. Harkin demonstrated that both C5 complement inhibition and, in another study, nitric oxide synthase inhibition (iNOS) provide effective protection in the serum and lung tissue by decreasing TNF-*α* [24]. In a review, Swartbol et al. reported that TNF-*α* was increased during surgery in almost all aneurism series and reached to the peak level in 6 hours, 15 times increased in the ruptured aneurysms versus the intact aneurysms, remained at peak levels for the postoperative 2 days, and increment persisted in those developed MODS [25].

İnterleukin-6 is responsible for the specific conditions in the inflammatory response of the host. It is produced as a response to the secretion of TNF-*α*, IL-1, or both cytokines. It is known that infusion of IL-6 alone does not generate any response. IL-6 initiates and stimulates oxidation in the neutrophils (respiratory burst), enhances ICAM-1 release from the endothelial cells, and enhances endothelial permeability. IL-6 is produced by hypoperfused skeletal muscle in the patients with peripheral artery disease and, in addition, it is systematically released in the reperfusion phase of aortic aneurysm surgery. In the present study, there was no difference between SIR and sham group in terms of IL-6 levels. There was no significant difference also in the SIR + EP group. Since IL-6 is generally secreted in the reperfusion phase being triggered by other cytokines, duration of reperfusion might have not been enough for this mediator to elevate. Kung et al. created a lung injury by LPS and determined decrease in IL-6 levels after 6 hours in the group received ethyl pyruvate [22]. It is known that IL-6 is increased in aneurysm ruptures and remains elevated when multiorgan failure is developed. Swartbol demonstrated that IL-6 reached to the peak levels within 6 hours to 7 days after surgical and endovascular treatment of intact AAA and RAAAs in various case series [25]. In the present study, absence of elevation in the SIR group as compared to the sham group could be explained by the fact that IL-6 has not been reached to the adequate serum levels yet in 2-hour reperfusion period. Probably, IL-6 levels could be measured more reliably in the postoperative surveys that would be performed in large animal models.

Various studies demonstrated that ischemia-modified albumin (IMA) is increased in the event of elevated free radical levels such as hypoxia or ischemia-reperfusion that tissues are exposed to sepsis, acute infection, advanced cirrhosis, end-stage renal disease, and advanced cancer [26]. IMA is ischemia-sensitive but not tissue-specific. Change in IMA in acute coronary syndromes has been investigated the most. Zhong et al. found a strong and independent correlation between IMA values and coronary artery disease, and Turedi et al. demonstrated that it is a prognostic marker after cardiopulmonary resuscitation in cardiac arrest patients [27, 28]. There are also various clinical studies demonstrating diagnostic value of IMA in the early stage of cerebrovascular events such as subarachnoid and intracranial hemorrhage or ischemic stroke, arterial occlusion, deep vein thrombosis, and mesenteric infarction. Gunduz et al. put forward the diagnostic value of IMA in acute mesenteric obstruction and in pulmonary embolus [29]. In the present study, IMA was significantly elevated in the S/C group, but the decrease in S/C + EP group was not found statistically significant. IMA has not been measured previously in the experimental model as we performed. Significantly, elevated IMA level in the SIR group confirms ischemia. Owing to overlapping other parameters, it can be used as an important marker that indicates ischemia in the experimental studies. The present study shows that IMA could be used in RAAA model. With regard to the lung's wet/dry weight ratio, severe pulmonary edema was observed in the S/C group which was substantially improved with ethyl pyruvate. In the ischemia-reperfusion studies, “Pulmonary Permeability Index” gives the most valuable information about the lung tissue. It is a quantitative method obtained by proportioning the measurement of radioactive iodine 125-labeled albumin in the serum and in the bronchoalveolar fluid [2]. In the present study, we could not perform this method used by Lindsay in the experimental model of RAAA but obtained information about pulmonary edema using the lung's wet/dry weight ratio used in many studies, in which the pulmonary edema had been demonstrated quantitatively. In the present study, lung's wet/dry weight ratio was 2 times increased in the SIR group versus the sham groups but significantly decreased in the SIR + EP group versus the SIR group. That is to say, EP significantly reduced the pulmonary edema. This condition, which is associated with endothelial permeability, can be explained by the suppression of lipid peroxidation; this is also confirmed by decreased lung MDA levels.

Blood gas analysis exhibits gas change at alveolar level and acid-base balance of the organism. Serum pH level was significantly decreased in the S/C group versus the sham group and acidosis occurred, base gap was developed, and a little improvement occurred in the group that received EP although being not significant. It is obvious that great changes in blood gas parameters could not be tolerated by the rats kept in spontaneous respiration in the room air and would result in death. One of the most important limitations of this experiment is leaving the rats in spontaneous respiration in the room air without intubating.

Histopathological examination revealed diffuse edema, leukocyte infiltration, and interalveolar and intra-alveolar hemorrhage in the lung tissue of the SIR group. Injury score of this group increased to 2.66 from 0.5 in the sham group. In the SIR + EP group, a decrease was observed in the intra-alveolar edema and leukocyte infiltration and injury score decreased to 1.44 from 2.66 in the SIR group. There was no difference in the sham + EP group as compared to the sham group; that is, EP alone has not cause any damage. There are many studies demonstrating that ethyl pyruvate acts as anti-inflammatory, antiedema, and antioxidant agent and treats organ injury in many experimental studies including hemorrhagic shock, ischemia- reperfusion, burn, sepsis, and acute necrotizing pancreatitis [30, 31]. However, studies that demonstrate its efficacy on distant organ injury such as lungs are quite limited. Karabeyoglu et al. demonstrated that ethyl pyruvate reduces lung injury seen after burn in the rat model [32].

The present study is a unique experimental model, which provides the simulation of both hemorrhagic shock and ischemia-reperfusion in the same experimental model. In this respect, we think that it will provide an important contribution to the literature.

Over the course of experiment, the sham groups maintained stable blood pressure showing minimal need for fluid replacement; however, in the S/C group, a remarkable hypotension and need for fluid replacement were observed which particularly increased as the aortic clamp was removed and reperfusion was started. This resulted from the vasodilation caused by vasoactive mediators and certain metabolites that joined the systemic circulation from the ischemic tissues during reperfusion phase, as well as from the increased vascular permeability. In the group that received ethyl pyruvate, inflammatory cell activation was suppressed and microvascular permeability decreased, more stable blood pressure levels were obtained, and the need for replacement was reduced. Pyruvate is a compound that structurally resembles lactate and is shown to help survival in hemorrhagic conditions due to its antioxidant and anti-inflammatory effects [31]. Tawadrous et al. demonstrated that impaired intestinal permeability and hepatic lipid peroxidation due to hemorrhage were significantly decreased in those which received ethyl pyruvate as compared to those which received traditional fluid replacement [33].

In conclusion, ethyl pyruvate prevents the lung injury created by experimental rat model of RAAA and shows this efficacy reducing the systemic and local inflammatory response by means of suppressing neutrophil infiltration and production of free radicals.

## Figures and Tables

**Table 1:** Schematic diagram of the interventions in each study group.

Groups	*n*	Shock 60 min	Treatment	Heparin 250 U/kg	(1/2) Blood resuscitation	Clamp 60 min	(1/2) Blood resuscitation	Reperfusion 120 min	Blood and tissue samples
Sh	6	−	RL	+	−	−	−	−	+
Sh + EP	6	−	40 mg/kg EP	+	−	−	−	−	+
S/C	9	+	RL	+	+	+	+	+	+
S/C + EP	9	+	40 mg/kg EP	+	+	+	+	+	+

Sh: sham; EP: ethyl pyruvate; S/C: shock/clamp; RL: Ringer's lactate solution.

**Table 2:** Biochemical parameters and histopathological scores in all groups (mean ± SD).

Parameters	Sh (*n* = 6)	Sh + EP (*n* = 6)	S/C (*n* = 9)	S/C + EP (*n* = 9)
MPO (ng/mL)	81.82 ± 34.21	96.91 ± 49.32	383.85 ± 38.19^a,b^	198.33 ± 69.16^a,b,c^
MDA (nmol/mL)	1.42 ± 0.43	1.45 ± 0.34	2.95 ± 1.45^a,b^	1.96 ± 0.61^a,b,c^
TNF*α* (pg/mL)	151.63 ± 30.64	147.52 ± 16.14	262.71 ± 18.24^a,b^	188.76 ± 56.55^a,b,c^
IL-6 (pg/mL)	164.62 ± 16.18	157.98 ± 19.93	196.53 ± 52.22	183.98 ± 56.75
IMA (ABSU)	0.42 ± 0.3	0.32 ± 0.21	0.84 ± 0.43^a,b^	0.74 ± 0.22^a,b^
Lung MDA (nmol/g)	454.29 ± 35.2	461.63 ± 51.26	522.5 ± 81.12^a,b^	499.14 ± 43.2^a,b,c^
Lung MPO (ng/mL)	5636.77 ± 404.7	5894.49 ± 971.17	6541.1 ± 613.4^a,b^	6019.12 ± 394.4^a,c^
W/D weight ratio	2.28 ± 0.18	2.21 ± 0.08	5.55 ± 0.45^a,b^	3.77 ± 0.74^a,b,c^
Histopathological score	0.5 ± 0.5	0.5 ± 0.4	2.66 ± 0.7^a,b^	1.44 ± 0.72^a,b,c^

Note: Sh: sham; EP: ethyl pyruvate; S/C: shock/clamp; MDA: malondialdehyde; MPO: myeloperoxidase; TNF*α*: tumor necrosis factor alpha; IL-6: interleukin-6; IMA: ischemia modified albumin; W/D ratio: wet/dry ratio.

^a^ *P* < 0.016 versus Sh.

^b^ *P* < 0.016 versus Sh + EP.

^c^ *P* < 0.016 versus S/C.

**Table 3:** Blood gas values.

	pH	PO_2_	PCO_2_	HCO_3_	BE
Sh	7.38 ± 0.04	115.75 ± 12.34	41.8 ± 5.7	25.45 ± 1.73	0.43 ± 1.38
Sh + EP	7.44 ± 0.08	107.43 ± 9.54	34.3 ± 11.6	23.51 ± 5.14	0.13 ± 2.49
S/C	7.20 ± 0.09^a,b^	79.88 ± 18.39	36.1 ± 7.05	15.4 ± 4.7	−9.38 ± 5.04^a,b^
S/C + EP	7.33 ± 0.07^a,b^	87.11 ± 14.29	36.9 ± 7.47	19.24 ± 2.88	−5.64 ± 2.8^a,b^

PaO_2_: arterial oxygen pressure; PaCO_2_: arterial carbon dioxide pressure; HCO_3_: bicarbonate; BE: base excess.

^a^ *P* < 0.016 versus Sh.

^b^ *P* < 0.016 versus Sh + EP.

^c^ *P* < 0.016 versus S/C.
